# National Health Insurance - knowledge, attitude and perceptions of speech-language therapists

**DOI:** 10.4102/phcfm.v17i1.4835

**Published:** 2025-05-27

**Authors:** Nomfundo Njilo, Andrew J. Ross

**Affiliations:** 1 Department of Family Medicine, College of Health Sciences, University of KwaZulu-Natal, Durban, South Africa

**Keywords:** NHI, speech-language therapy, KwaZulu-Natal, healthcare services, discourse analysis

## Abstract

**Background:**

The South African government signed the National Health Insurance (NHI) Bill into effect on 15th of May 2024 to ensure that all citizens have access to high-quality healthcare, regardless of their financial situation. While this initiative will impact all healthcare professionals, there is limited information on how speech-language therapists (SLTs) perceive its implementation in South Africa.

**Aim:**

The study aimed to explore the knowledge, attitudes and perceptions of SLTs in the public and private healthcare sectors related to service provision regarding the implementation of the NHI.

**Setting:**

This study was conducted virtually via Zoom (Zoom Video Communication, San Jose, California, United States) and Teams (Microsoft Teams, 2017) with SLTs in their respective settings in South Africa.

**Methods:**

The descriptive, qualitative research design involved virtual semi-structured interviews with 10 SLTs. NVivo software (QSR International, Victoria, Australia) was used to analyse the data, as guided by Tesch’s content analysis method.

**Results:**

Eleven sub-themes emerged related to the three themes of knowledge (4 sub-themes), attitudes (4 sub-themes) and perceived impact (3 sub-themes) of NHI implementation on SLT services.

**Conclusion:**

The study highlights SLTs’ knowledge, gaps and concerns about the impact of NHI implementation on their profession, emphasising the personal and professional areas that need to be addressed for its successful rollout.

**Contribution:**

Understanding SLTs’ opinions will help address their concerns during the planning stages of integrating them into the NHI. This will lead to an equitable distribution of sufficient practitioners and ensure that many people benefit from its implementation.

## Introduction

Speech-language therapists (SLTs), also known as speech pathologists, play a crucial role in assessing and treating communication, speech, language, cognitive-linguistic and swallowing disorders across all age groups, which are delivered in both public and private healthcare settings in South Africa (SA). After completing their academic training and a compulsory year of community service, SLTs can choose to work in diverse healthcare environments, including private practices, hospitals, community-based centres and educational facilities. South Africa has a disability rate of 7.5%, with 1.8% of the population experiencing varying degrees of communication difficulties, including issues with speech, language and voice.^1^ While it is challenging to estimate the exact incidence and prevalence of individuals with swallowing and communication disabilities, research suggests that nearly 49% of those seeking rehabilitation services have communication challenges.^2^ Among patients requiring speech therapy, many present with complex multimorbidities, necessitating ongoing care post-hospital discharge.

This underscores the need for step-down rehabilitation facilities and readily accessible outpatient SLT services, in both the private and public sectors, to improve health outcomes.^3^

Despite the demand for SLT services, access to care is unequal across SA, with the majority of patients only being able to access such services at public sector hospitals through referrals from primary healthcare (PHC) facilities. A significant portion of SLTs are concentrated in private practices in urban areas, while most of SA’s population (80%) depends on the public healthcare system.^4^ Approximately 20% of SLTs are employed in the public health sector, and their distribution is often uneven, with some public hospitals having only one therapist (often a community service therapist) available to cover all those who need treatment.^4^ The lack of services at the lowest level of care, the PHC clinic, forces many patients to travel long distances to the relevant district hospital, often using public transport, and incurring additional costs.^4^

The Health Professions Council of South Africa (HPCSA) has reported a critical shortage of SLTs in the public sector, leading to significant delays in treating patients with dysphagia (swallowing problems) and communication disorders. Current therapist-to-patient ratios fall far short of international benchmarks, such as the Royal College of Speech and Language Therapists’ recommendation of one SLT per 10 patients. As a result, SLT positions are limited, and speech and communication disorders are often deprioritised in the public healthcare system. This is compounded by challenges such as inadequate healthcare financing, poor leadership, incomplete data and insufficient human resources.^3^ As most SLTs are employed in the private sector, particularly in urban areas, studies suggest that more incentives are necessary to encourage therapists to move into the public sector, specifically in rural and under-serviced areas.^4^

To achieve universal health coverage (UHC) and equitable access to quality healthcare, the SA government introduced the National Health Insurance (NHI) Bill in May 2024. The NHI aims to eliminate financial barriers to healthcare, allowing individuals without private medical coverage to access services at private practices, which will be reimbursed through an NHI billing system. Funding for the NHI will come from mandatory contributions from individuals earning above a certain income, government allocations and partnerships with the private sector. Contributions to private medical aid schemes will be redirected to the NHI, with private sector services being available only for those not covered by the NHI.^5^ However, private practitioners will have the option not to participate in the NHI.

The proposed NHI system in SA aims to integrate the private sector into a publicly managed healthcare framework to improve service availability in underserved areas, particularly rural regions. Central to this initiative is the Contracting Unit for Primary Healthcare (CUP), a government-funded agency that will oversee healthcare service contracts and payments. Apart from confirming the establishment of the NHI services, the Act includes plans for one CUP in each province. These units will focus on managing primary healthcare services through agreements with public sector district hospitals, clinics, ward-based outreach teams and private providers and monitoring the distribution of funds to healthcare service providers. A significant shift in this model is the move away from the traditional fee-for-service reimbursement system, which compensates medical professionals per consultation or treatment.

The Department of Health will manage the NHI and procure services from public and private sector providers. Practitioners contracted into the scheme, including SLTs, will be compensated through various payment models, raising concerns about service delivery and resource allocation. South Africa has only about 1638 registered SLTs, far below the estimated 5000 needed to meet the anticipated demand, particularly in the overburdened public sector.^6^ The country is currently in the third phase of the NHI) rollout (2023–2026), which aims to strengthen health systems and mobilise resources. Following the establishment of policy frameworks, the *NHI Act of 2023* mandates payments and emphasises primary healthcare access. However, since its signing in March 2024, the initiative has faced criticism because of various challenges, such as under-resourced public institutions, rising disease burdens and concerns about its financial and logistical feasibility.^7^ Many healthcare professionals, including SLTs, are uncertain about how the NHI will impact service delivery, professional autonomy and resource allocation. The official government website indicates that the NHI aims to provide healthcare for all, underpinned by the SA government’s legislative mandate to implement universal health coverage for every one.^8^

This study aimed to explore the knowledge, attitudes and perceptions of SLTs currently working in both the public and private sectors regarding the implementation of the NHI, about how their profession and service delivery will be impacted. Understanding the perspectives of SLTs, who are integral to rehabilitation services, is crucial to ensuring the successful integration of speech therapy into the NHI framework.

## Research methods and design

### Study design

A qualitative study design with an exploratory, descriptive approach was used to generate unique insights related to the SLT knowledge, attitudes and perceptions regarding the NHI.^9^

### Setting

This study was conducted virtually via Zoom (Zoom Video Communication, San Jose, California, United States) and Teams (Microsoft Teams, 2017) with SLTs in their respective settings or provinces in South Africa practising in the private or public sector. This required participants to have access to video conferencing technology and devices. Zoom and Teams were deemed the most suitable platforms to conduct interviews for this study as they were the platforms most participants were familiar with.

### Study population, sample size and sampling strategy

The study population was SLTs registered with the HPCSA who were working in the public or private sector in South Africa in 2024 with at least 2 years of experience post community service. Practitioners who were dually qualified as both SLTs and audiologists were excluded. Convenient sampling was used to recruit participants with those who responded to the poster invitation shared in closed Facebook and WhatsApp groups for SLTs and who met the inclusion and exclusion criteria invited to participate in the study. A sample size of six to nine participants was initially considered adequate for this study.

### Data collection

Once participation was confirmed and participants met the inclusion and exclusion criteria, semi-structured interviews were arranged at a mutually convenient time. A semi-structured interview guide was developed using the four phases of the Interview Protocol Refinement (IPR) framework to ensure the reliability of the guide and the depth of qualitative data gathered.^8^ The interview guide was reviewed and refined after feedback from two experts: expert 1, an SLT and certified lactation consultant with a master’s degree and public health sector experience, and expert 2, a healthcare researcher with 20 years experience developing research protocols. Two SLTs piloted the guide, and further adjustments were made based on their feedback. The guide consisted of six broad questions aligned with the study’s objectives, focussing on participants’ knowledge of the NHI as well as their attitudes to and perceptions of its impact on SLT service provision. Probes and follow-up questions were included to elicit more in-depth responses. Interviews were conducted individually via Zoom and audio-recorded with consent. Zoom’s automated transcription feature was utilised, after which the first author verified and corrected the transcriptions.

### Data analysis

Data were analysed using Tesch’s open coding method, following Creswell’s (2018) content analysis approach.^9^ The first author read through all transcriptions repeatedly to gather a deeper understanding of the data and to identify recurring patterns. The first author systematically coded the transcripts, identifying key concepts and categorising them into preliminary themes using NVivo software (QSR International, Doncaster, Victoria, Australia). Expert 1 followed the same steps as the first author to ensure trustworthiness of results, after which a meeting was virtually held to discuss discrepancies and tabulate final themes and sub-themes. Data were analysed after the sixth interview and interviews continued until data saturation was reached, which was after interview number 10, as no new themes emerged. Detailed descriptions of the participants’ context and experiences have been provided, allowing readers to assess the applicability of the findings to similar settings or populations.

### Reflexivity

The interviews were conducted by the first author, a South African practising SLT with 3 years of experience including 1 year of community service, and 2 years in private rehabilitation and special education settings. The second author is a South African family physician with extensive expertise in qualitative research. Although the first author has limited experience in qualitative research, support and mentoring were provided by the second author. There was no relationship between researchers and participants. Efforts were made to minimise researcher bias by regularly reflecting on the data and the analysis process. The use of an independent coder helped ensure that interpretations of the data were grounded in the participants’ accounts rather than the researchers’ perspectives.

### Ethical considerations

Interested individuals were given an information sheet and an informed consent form, which they signed and returned via email before scheduling an interview. Ethical clearance was obtained from the University of KwaZulu-Natal Humanities and Social Sciences Research Ethics Committee (HSSREC) (No. HSSREC/00006642/2024).

## Results

A total of 10 SLTs participated in the study (Table 1), their current work settings varying, with some providing inpatient care (5 participants), outpatient care (6 participants) and others offering services in home-based residential settings (3 participants). Four participants worked in the private sector and six in the public sector, with most of the clients seen in private practice having medical aid insurance, while those attending public healthcare facilities did not.

**TABLE 1:** Description of participants.

Participant no.	Age (years)	Gender	Experience (years)	Sector currently working In	Location
1	24	Female	2	Public	Rural KwaZulu-Natal
2	25	Female	3	Public	Rural Eastern Cape
3	25	Female	2	Public	Rural Mpumalanga
4	26	Male	3	Public	Rural KwaZulu-Natal
5	24	Female	2	Private	Urban Free State
6	26	Female	4	Private	Urban Gauteng
7	23	Female	2	Public	Rural KwaZulu-Natal
8	27	Female	5	Private	Urban KwaZulu-Natal
9	27	Female	5	Public	Urban Gauteng
10	35	Female	10	Private	Urban KwaZulu-Natal

Table 2 shows the themes and associated sub-themes related to the knowledge, attitudes and perceptions of SLTs in the public and private sectors towards implementing the NHI.

**TABLE 2:** Summary of themes and sub-themes.

Themes	Sub-themes
1 Knowledge of the NHI	1.1 Information needs 1.2 Structured funding system 1.3 Equitable access to healthcare
2 Attitudes to NHI	2.1 Private practice establishments 2.2 Continuation of care
3 Perception of NHI on SLT services	3.1 Reduced financial cost for patients 3.2 Increased administrative duties 3.3 Need for more staff

NHI, National Health Insurance; SLT, speech-language therapist.

### Theme 1: Knowledge of the National Health Insurance

The participants indicated that they had limited knowledge about the details surrounding the implementation of the NHI, specifically as it related to SLT services. Without specific knowledge, their limited understanding resulted in three sub-themes: information needs, structured funding system and equitable access to healthcare.

#### Sub-theme 1.1: Information needs

The participants admitted that their limited knowledge meant they had information gaps regarding the NHI and how it would function once implemented, specifically regarding SLT services:

‘I am not sure what it specifies about speech therapy, but I think they must specify which services will be provided. So, we must not just say people can access the services; they need to specify which services, how long, how frequent, etc.’ (P1, Female, 24 years, Public)

‘I have not looked at the specifics or anything like that.’ (P10, Female, 35 years, Private)

‘I know very little about the NHI.’ (P9, Female, 27 years, Public)

#### Sub-theme 1.2: Structured funding system

Participants had a limited understanding of how the NHI would be funded and described the source of funding as a system in which there will be a central fund to which everyone who is employed contributes. Service providers will then claim from this central fund to pay for healthcare services:

‘It is like an insurance fund, where the government pays for healthcare services and provides healthcare to all South African residents.’ (P7, Male, 23 years, Public)

‘What I heard is that they are going to raise funds through tax. They will raise funds through personal income taxing, general taxing and the other taxes that I need help remembering. So yeah, the upper and middle classes will be paying for NHI.’ (P9, Female, 27 years, Public)

‘As middle-class citizens, we find ourselves in a situation where our financial contributions primarily support initiatives that may not directly benefit our own families. For instance, while we collectively fund the National Student Financial Aid Scheme (NSFAS), our children often do not qualify for its assistance, leaving us to shoulder the burden of education costs alone. Similarly, the National Health Insurance (NHI) is funded through our taxes, yet it appears to prioritise the needs of the unemployed or lower-income groups.’ (P9, Female, 27 years, Public)

#### Sub-theme 1.3: Equitable access to healthcare

Many of the principles guiding the NHI are similar to those of the primary healthcare system (PHC), such as providing quality healthcare to individuals and families through full participation and at a cost people can afford. Participants shared that the NHI will provide quality healthcare, including SLT services, to all citizens, regardless of their economic status and geographical areas. They indicated the following:

‘Certain patients would be able to access services they previously could not access privately because they would have to pay or have medical aid. It is more of a method to ease the burden on the public healthcare system.’ (P1, Female, 24 years, Public)

‘We will be able to get healthcare services. Whether you are rich or poor, we will all get the same healthcare services.’ (P2, Female, 25 years, Public)

‘It will also increase access to speech therapy services. For example, we are short-staffed and whenever one of us goes on leave, we cancel our OPD patients. So, our patient does not receive services for that week. If the NHI gets implemented, we will have more speech therapists in the public sector and might not need to cancel the services because one of the speech therapists is on leave.’ (P9, Female, 27 years, Public)

### Theme 2: Attitudes to the National Health Insurance

The attitude of participants towards the NHI related to the roles they saw SLTs playing as service providers under the new healthcare management system, with two sub-themes emerging: private practice establishments and continuity of care.

#### Sub-theme 2.1: Private practice establishments

There was some ambivalence about the effect that the NHI would have on private practices. Some SLTs felt that they would likely experience difficulty setting up a new department in a private hospital or a new private practice under the NHI framework, which will require acquiring the relevant licenses to be integrated into the NHI. However, others felt that the NHI presented a business opportunity for SLTs:

‘NHI, for me, has a business aspect. It makes you want to start a business or open a private practice. So now, we are all developing an interest in owning practices instead of just running a practice on a Saturday while you have a government job Monday to Friday.’ (P4, Male, 26 years, Public)

‘If I’m accredited by the NHI as a contracting unit, anybody can come and access services from me, from my private practice. So, I think, to sum it up, it’s just the uncertainty or the reality of how it’s going to play out.’ (P4, Male, 26 years, Public)

#### Sub-theme 2.2: Continuation of care

The participants’ attitude was that under the NHI, their roles would not change, as they would continue providing the same services, irrespective of whether they worked in the public or private sectors. In the private sector, their client base may change, with the inclusion of those traditionally served by the public sector, but the nature of their services would remain the same:

‘Just like now, our role will not change. Okay, so we will have to give the services to the public, whether you are in private or public. You must render quality services.’ (P3, Female, 25 years, Public)

‘My role as a speech therapist within the NHI will be to see patients, treat them well and offer speech therapy services.’ (P5, Female, 24 years, Private)

‘One key advantage is that everyone would have access to affordable speech therapy services, addressing the issue of high costs that currently limit access for many people. This would ensure equal access and increased frequency of treatment and better outcomes for individuals in need.’ (P8, Female, 27 years, Private)

### Theme 3: Perception of the National Health Insurance on speech-language therapy services

The SLTs’ perceptions regarding SLT service provision for the NHI were indicated by three sub-themes: reduced financial costs for patients, increased administrative duties and need for more staff.

#### Sub-theme 3.1: Reduced financial cost for patients

Some participants felt that implementing the NHI would minimise the costs that families would incur in accessing SLT services, as they would be able to use private practitioners who were close by and not have to travel to a public sector hospital, which could be far away. One participant noted:

‘The advantage is that I am in a rural area, and it is difficult for people to access a speech therapist. They do not have money to travel far to the hospital because it is so costly. After all, it is him and the child, or her and the child. Therefore, if they can access the (private) services, it means that in their area, there are better outcomes for intervention, and they spend little money.’ (P2, Female, 25 years, Public)

#### Sub-theme 3.2: Increased administrative duties

Some participants felt that there would be more administrative responsibilities and challenges with the implementation of the NHI, specifically for those in the private sector, as it would require submitting claims to ensure payment, which could be delayed if the system was inefficient:

‘I think it’s going to mostly affect private practice owners— they’ll be the ones disadvantaged. Even now, when someone uses the private healthcare system, there are already problems, like government medical aid funds not paying private doctors on time. I think this will bring in a lot of admin work, and that’s going to affect how many patients they can see in a day. Now they’ll have the extra task of filling in forms and submitting them to the state to get paid after seeing each patient.’ (P1, Female, 24 years, Public)

#### Sub-theme 3.3: Need for more staff

The SLTs felt that there were not enough practitioners in the public sector in SA to meet the current demands, which affects timeous and appropriate interventions, these being important principles of the NHI. Enabling patients to access private SLTs might take the pressure off some areas, but as there are very few SLTs in rural areas, many more will be needed to provide services at public sector hospitals:

‘Another important thing is hiring more speech-language therapists. We serve a large number of communities, and we can’t expect just one or two therapists to cover the entire city without being overworked.’ (P9, Female, 27 years, Public)

‘One does not have sufficient speech therapists to offer the services, but now everybody will have the right and the means to access a service. I need to ensure I have the people who can offer the service.’ (P4, Male, 26 years, Public)

## Discussion

Most participants had little knowledge of the NHI and lacked a clear understanding of how it would function, indicating the need for further information. This finding is consistent with another study conducted in SA, where 60% of community pharmacists described their knowledge of the NHI as satisfactory, while 40% highlighted needing more information.^10^ While the gazetted services that will be offered under the NHI include rehabilitation health services, which encompass SLT, there is little information about how its implementation will affect health practitioners in private practice.^11^ The fact that participants knew very little about how it affects their profession is concerning, as it means that decisions are being made to proceed with the process without consulting the practitioners who will have to implement it. This highlights the need for much greater information dissemination to health practitioners in general and SLTs in particular, given their shortage in the country, which has implications for service delivery.

Some participants said that the NHI would be funded through taxes and managed by a centralised government payment system, to which tax-paying citizens would contribute through various means of revenue collection. According to the *NHI Act 20 of 2023*, South Africans will no longer need to make direct contributions to a private medical health insurance scheme, as all residents will be covered by the NHI. The funds that the government appropriates from what it generates each year will be the main source of income and will come from general tax revenue, payroll tax, a personal income tax surcharge and the reallocation of funds for medical scheme tax credits.^12^

The National Health Services (NHS) in the United Kingdom is also funded through taxation and provides services to over a million patients every 36 h, with issues such as dentistry and optometry being excluded, which means that people need to pay for those services.^13^ However, the UK has a large tax base, despite its population being a similar size, with 68.2 million people paying personal taxes and millions contributing to their health system recently, with reports indicating that the NHI model in the UK is not sustainable for SA.^14^

In 2018, Zambia, a lower-middle-income country in Southern Africa with a population of 18 million, enacted the *NHI Act* to promote universal access to quality health services.^10^ The package offers a wide range of services, including outpatient and inpatient care, surgical procedures, maternity and neonatal support, eye and dental health services, prescription medications and physiotherapy. According to current regulations, employees must contribute 1% of their monthly salary, with employers matching this amount. For self-employed individuals and those in the informal sector, the contribution is also 1% of their declared monthly income, with a minimum contribution set at 60 kwacha (approximately R39.28 South African).^10^ The introduction of the National Health Insurance Scheme (NHIS) has improved access to healthcare for the Zambian population, especially for those who are poor and vulnerable. Nonetheless, despite its successful rollout, the NHIS encounters various challenges, such as limited coverage, low quality of care, corruption, ineffective governance, inadequate stakeholder involvement, unclear policy concepts, solid political influence and insufficient funds.^11^ Given the lack of details about how the funding will work in SA, the reliance on the tax base was a concern for the participants, given the high unemployment rate with the middle and small upper classes having to carry the main financial burden of funding the initiative.

Some were aware that the aim of the NHI was to ensure equitable access to healthcare services for everyone, including SLT services. However, the challenges of the unavailability and inaccessibility of such services were of concern among the professionals, given the lack of sufficient practitioners to staff the public sector hospitals. The *NHI Act* places a strong emphasis on equitable access, this being one of the main reasons for its implementation, to equalise the disparity in services between the private and public sectors, both in terms of number and distribution. ‘The *National Health Insurance Act 20 of 2023* intends to achieve universal access to quality healthcare services in the Republic in accordance with section 27 of the Constitution’.^15^ However, without clear guidelines about what services would be provided, it was difficult for participants to conceptualise how these might be delivered, given the current shortage of SLTs.

The SLTs had ambivalent attitudes about establishing private practices, with concerns about difficulties related to opening new facilities and whether that would need government approval. Some participants felt that it was unclear how private practice establishments would contractually work with the government and acknowledged the need for more contractors to meet the needs of a greater population. To be accredited as a contractor, providers must meet specific performance criteria and be registered with recognised health professional councils. A legally binding contract would be established between the NHI and the providers for the delivery of primary healthcare, emergency medical and hospital services. To receive reimbursement for services rendered, providers must submit certain required information to the fund. The NHI official government website indicates that it aims to ensure that all South Africans access healthcare services through public or private facilities that are nearest them, with little information about how this will be done.^11^

The participants’ attitudes were that their role in the NHI would not change and would continue to be centred on the continuation of care. They felt that having access to additional SLTs would improve compliance with treatment programmes, as patients would be able to see them more regularly if they lived closer. As the therapists in the private practices would be there long term, they would be able to build relationships with patients if their treatment required care for many years, which was not possible in public sector facilities, which often rely on community service students who change yearly. Having SLTs close at hand would mean that when problems arose, patients would not need to wait for their next clinic appointment to get it resolved but could contact the practitioner and have the issue addressed, which could prevent complications.

The participants perceived that implementing the NHI would reduce the costs for patients to access SLT services because of their being closer to private practices, should they be available. In a social media-based study conducted in SA in 2014 with Mxit, the participants regarded the public health sector as important for poor people who could not afford to pay for health services but also described challenges when patients needed to pay for services at public healthcare institutions.^16^ The NHI’s implementation should result in universal coverage to give patients access to (reasonably) affordable SLT services, with no costs for those who cannot pay and payment being required for those who can. An outline of the new health system’s financing is given in the NHI Green paper, with monies distributed based on need. The financial system’s conceptual division comprises three interconnected functions: revenue collection, fund pooling and purchase and service providing.^11^ Current payment rates are set by medical aids and the HPCSA, with no clarity on what body will set the new rates under the NHI, and if it will be worth joining from a financial perspective, even in underserved areas, where the client base is likely to make the business sustainable.

A study conducted in SA regarding the role of pharmacists during the implementation of the NHI in 2021 revealed that participants perceived that their role would be reduced to pill dispensers with lots of administrative work to submit to get paid.^2,17^ There are no clear guidelines on how the integration of the private sector into the NHI will work, what the administrative requirements will be to access payment for the clients who are seen and what payment structures will be available, which has made it difficult for SLTs to form any opinions about whether or not they would want to participate in such a system.

All the participants agreed that more SLTs need to be trained to not only fill the vacancies in the public sector but to open private practices in those areas where no state-funded facilities are located. The 2017 White Paper on the NHI in SA highlights several key initiatives aimed at enhancing healthcare delivery through targeted teams and a focus on human capital.^5^ This would involve a number of measures: (1) training and education to ensure that healthcare workers are adequately trained to meet the needs of the population. (2) Retention strategies: developing initiatives to keep skilled professionals in the system, especially in underserved areas. (3) Skill development: continuously enhancing the skills of existing healthcare workers to adapt to evolving healthcare challenges. (4) Importance of human capital: as highlighted by the World Health Organization (WHO), having a well-trained and sufficient workforce is essential for a successful and sustainable public health system. These measures not only improve service delivery but also enhance health outcomes for the population.^5^

How the shortages of staff will be addressed is not indicated in the *NHI Act*, with no comments being made about training those disciplines that are in short supply or attracting those from other countries.^6^ The lack of sufficient SLTs in both the private and public sectors might be exacerbated with the implementation of the NHI, with more practitioners needing to be trained to meet the increased demand for services. South Africa is likely to have a critical shortfall of speech therapists in 2030, which means that policymakers will have to carefully examine issues surrounding the current framework regulating training of these and associated professionals to respond adequately to future requirements).^6^

### Limitations of the study

The study relied on SLTs’ perceptions, which may be subject to recall limitations and personal bias. Convenience sampling was used to recruit participants, which may have biased the results as those with strong opinions about NHI may have been more likely to volunteer to participate in the study. Participants may therefore not be representative of the wider SLT community and may not have expressed the opinions of other SLTs if purposeful sampling had been employed. In addition, only one male participant volunteered to participate in this study, which may also have biased the results. Most of the participants had worked in the public sector for no more than 4 years, which means that they may have limited experience, and their responses may not reflect those who have worked longer in the public sector. Differences in socio-economic conditions and institutional support across different private practices might also have influenced the participants’ perceptions of the NHI and how it will function, and these factors might not be fully captured in the study. However, as this is an exploratory study, insights gained from across a range of SLTs working in different provinces, in public and private settings, give valuable insights into their current perception of NHI.

### Implications

Speech-language therapists need to be prepared for potential changes in service delivery, requiring professional development opportunities that focus on adapting to the NHI model of service delivery. The perceived increase in demand for SLT services under the NHI requires caseload management adjustments, such as scheduling and service prioritisation.

The national department of health needs to provide educational workshops about how the NHI will impact rehabilitation professionals such as SLTs. Comparative research with other health systems that have implemented similar universal healthcare models could provide insights into the best practices for SLTs. Further studies should explore the long-term impact of the NHI implementation on SLT service delivery, professional satisfaction and patient outcomes.

## Conclusion

The results showed that for the NHI to be implemented as intended, with citizens having access to good quality health services, including SLT, more information needs to be made available about how the programme will work, and if this has not been established, those participating need to be consulted to ensure their support. The cost implications for both the providers and users need to be clarified, along with the availability of sufficient practitioners to ensure the availability of services across the country. As part of the team of rehabilitation therapists, SLTs provide essential services for people with some form of disability or limitation, and their services are often required on an ongoing basis to enable affected people to enjoy a good quality of life. This will only be possible with well-defined policies, regulations and procedures on how the NHI will function, as well as sound administration systems, and a clear indication of what services will be offered, including those by SLTs.
